# Advancing non-human primate welfare: An automated facial recognition system for unrestrained cynomolgus monkeys

**DOI:** 10.1371/journal.pone.0319897

**Published:** 2025-04-08

**Authors:** Yosuke Numata, Brian Sumali, Ken’ichiro Hayashida, Hideshi Tsusaki, Yasue Mitsukura

**Affiliations:** 1 IKI Japan, Shinjuku, Tokyo, Japan; 2 Faculty of Science and Technology, Keio University, Yokohama, Kanagawa, Japan; 3 Drug Safety Research Laboratories, Shin Nippon Biomedical Laboratories Ltd., Kagoshima, Kagoshima, Japan; 4 School of Medicine, Keio University, Shinjuku, Tokyo, Japan; University of Baghdad, IRAQ

## Abstract

Cynomolgus monkeys (*Macaca fascicularis*) are vital in biomedical research, particularly for drug development and studying neurological diseases. However, accurately identifying individuals in group housing environments remains a significant challenge. This paper presents a near real-time facial recognition system tailored for cynomolgus monkeys, utilizing a fine-tuned Detectron2 model for face detection, followed by eigenface-based classification with Support Vector Machine (SVM) and radial basis function (RBF) kernel. The system achieved an accuracy of 97.65% in 10-fold cross-validation and identified individuals in under 1 minute under ideal conditions. This method eliminates the need for invasive identification techniques, potentially reducing stress and improving animal welfare, and has the potential to reduce the need for individualized housing or specialized enclosures. Additionally, as the system reduces the time and labor required for identifying monkeys, it might benefit research facilities with high turnover rates. This method could improve identification in non-human primate research while minimizing stress associated with traditional techniques.

## Introduction

Cynomolgus monkeys (*Macaca fascicularis*), due to their physiological and genetic similarities to humans, play a crucial role in biomedical research, particularly in the development of pharmaceuticals and the study of neurological diseases. However, one of the significant challenges in conducting research with these primates in group housing environments is the accurate and efficient identification of individual monkeys. Traditional methods, such as physical tagging or manual observation, are not only invasive but also prone to errors and can significantly stress the animals, potentially influencing the research outcomes. In addition to these concerns, our facility for cynomolgus monkeys has annual turnover of approximately 3,000 monkeys with hundreds of enclosures. Moreover, individual monkeys were transferred within one month of arrival. As such, manual identification by human memory is practically impossible. and there is a critical need for fast and accurate identification that requires only as few learning images as possible – such as using images taken only during their registration on the first arrival at the facility.

Facial recognition has undergone significant development from its early stages to current advancements. The earliest techniques, like template matching, emerged in the 1960s, but they relied heavily on precise positioning and suffered from high computational costs due to their pixel-by-pixel approach [1–8]. As a more advanced method, eigenfaces (EF), introduced in the 1990s, brought a revolutionary approach by using Principal Component Analysis (PCA) to reduce dimensionality in facial datasets [9]. EF are computationally efficient compared to earlier methods, reducing memory usage by focusing only on essential facial features. Despite these benefits, EF also have limitations, particularly when dealing with variances in lighting, pose, and facial expressions, which are critical for real-world applications. Subsequent techniques like Fisherfaces [10] and Local Binary Patterns (LBP) [11,12] built on the EF method, addressing some of its weaknesses by being more robust to lighting changes and facial expressions. In recent years, deep learning methods, especially Convolutional Neural Networks (CNNs), have transformed facial recognition by improving accuracy and robustness [13–17]. CNNs leverage large datasets and complex architectures to learn detailed facial features, achieving state-of-the-art accuracy. Furthermore, integrating attention mechanisms with CNNs has enhanced feature extraction by focusing on the most relevant parts of the image, improving recognition performance even in challenging conditions [13]. Despite the higher accuracy, CNNs demand significant computational resources and memory, as they rely on large datasets and complex architectures.

Recent advancements in computer vision and machine learning have opened new avenues for non-invasive identification for both humans and animals. These computer vision-based systems are very helpful for identifying mammals, marine animals, birds, and even small animals like insects, with each category posing unique challenges [18–23]. Mammals such as primates and elephants are often subjects for individual recognition, which is essential for tracking their health, migration patterns, and behavioral changes [24,25]. In marine environments, species like dolphins and whales are identified based on unique markings on their fins or flippers, often requiring underwater image recognition to overcome low visibility and lighting fluctuations [26,27]. These advances help conservationists monitor specific animals within populations where each member’s health and behavior can impact the group.

Studies have shown promising results in applying advanced image processing and machine learning techniques to primate species [25,28–35]. These methods, despite their varying degrees of success, highlight the potential of applying sophisticated image processing and machine learning algorithms for primate identification, offering a less invasive, more efficient, and potentially more accurate alternative to traditional methods. Unfortunately, studies focusing on cynomolgus monkeys remain limited in number and more papers can be found that focuses on chimpanzees [28–30], gorillas [28,36], and macaques [37].

Based on the needs from our housing facility and building from aforementioned studies, our research explores a novel automated facial recognition system specifically designed for cynomolgus monkeys. This system leverages a fine-tuned Detectron2 [38] framework for accurate face detection, coupled with an EF-based classification approach that utilizes traditional machine learning classifiers, with the Support Vector Machine (SVM) with radial basis function (RBF) kernel showing optimal performance. This study contributes to the growing body of knowledge on primate facial recognition and addresses the unique challenges posed by the cynomolgus monkeys’ facial features, group housing environments, along with the need for near real-time processing capabilities.

This paper details the development and testing of our facial recognition system, focusing on its accuracy and efficiency in biomedical research settings involving cynomolgus monkeys. Through rigorous testing and comparison with existing methods, we evaluate the system’s strengths and weaknesses, providing a clear assessment of its performance. This study contributes to the understanding of the system’s applicability in research methodologies, with a focus on its current capabilities and limitations.

## Materials and methods

This study employed a three-phase approach to develop and evaluate a facial recognition system for cynomolgus monkeys. For the first phase, eight individually housed male monkeys were used as the subject. The Viola-Jones method [39] commonly utilized for human facial detection system were unable to detect the faces of these cynomolgus monkeys unlike rhesus macaques [33], and needed to be replaced with Detectron2, an object-recognition Convolutional Neural Network (CNN), which were fine-tuned via our manually labeled images. Various feature extraction and machine learning techniques were then applied, including Local Binary Patterns Histogram (LBP-H) [11,40], Eigenface (EF) [9], and Support Vector Machine (SVM) with various kernels, among others, to identify the most effective combination. The aim of this phase is to determine the possibility of automatic identification of individual cynomolgus monkey faces.

The second phase focuses on expanding the automatic identification to images with varying lighting, quality, and orientation. Still images of individual monkeys under anesthesia and video recordings of freely moving monkeys inside the group cage were taken. Only the best combination of feature extraction and classification were utilized to check the feasibility for the final phase: implementation and testing of the real-time recognition system against freely moving monkeys inside the group cage.

### Equipment

The recordings for all datasets utilized a Sony Handycam FDR-AX45A, while real-time testing during the third phase used a Logitech c930s external webcam as an input. Lighting was uncontrolled to simulate natural conditions, both in the training and testing set. The first and second phase training/validation was performed on a typical computer: OS Windows 10 x64, Intel Core i7-10700 @ 2.90 GHz, DDR4 32GB @ 2400MHz, NVIDIA GeForce RTX2060 SUPER 8GB, while real-time implementation and testing were conducted on a Dell Alienware X16 laptop. Matlab R2022a and Python 3.7 were utilized for analysis-validation and real-time system implementation-testing, respectively.

### Animals

The study involved two groups of cynomolgus monkeys under different housing conditions. The first group comprised eight male monkeys, housed individually in environments with a consistent 12-hour light/dark cycle. These animals were identified by tattoos on their chests and were utilized only during the first phase of the study. The second group was accommodated in three large facilities, each containing 12 to 17 monkeys (housing 1: 12 females, housing 2: 16 males, housing 3: 17 males). This group was exposed to partial natural light supplemented with artificial lighting on a 12-hour cycle and used exclusively for the second and third phases of the study, with identification tattoos on their inner thighs. The care and use of the animals in this research were overseen and approved by the Institutional Animal Care and Use Committee of Shin Nippon Biomedical Laboratories, Ltd.

Group 1 consisted of eight male cynomolgus monkeys housed in individual cages. They were fed twice daily, in the mornings and evenings, with a diet that included fruits and vegetables, while water was available *ad libitum*. Their environment was enriched with plastic balls and plastic toys to encourage play and mental stimulation. To alleviate potential suffering caused by stress from video recording, the recordings were performed by caretakers familiar to the monkeys, which helped reduce anxiety. Health monitoring for this group was conducted once daily by caretakers, who assessed mood and behavior along with checking physical injuries. Additionally, an annual in-depth health checkup was performed by a veterinarian. The animals were maintained in captivity under humane conditions until the end of their natural lives.

Group 2 comprised three separate housings for group-housed cynomolgus monkeys: Housing 1 with 12 females, Housing 2 with 16 males, and Housing 3 with 17 males. They shared a feeding regimen of twice-daily meals consisting of fruits and vegetables and had water available *ad libitum*. Environmental enrichment for this group included climbing structures such as swings, ropes, and ladders, as well as plastic toys, to promote physical activity and social interaction. To mitigate stress, close-up photography, which required anesthesia with ketamine, and catching monkeys (during real testing) were performed by experts to minimize monkey capture time and handling. Health monitoring was similar to Group 1, with daily assessments by caretakers (measures: mood, behavior, hair loss, and wounds) and an annual veterinarian checkup. These monkeys were also maintained in captivity under humane conditions until the end of their natural lives.

### Dataset collection

Two datasets were generated during this study – one for each animal group described in the previous section. The dataset for the first phase originated from the eight individually housed monkeys. Videos were filmed using the Sony Handycam, then resampled at 10 FPS. Detectron2 processed these images to isolate faces, followed by manual inspection to ensure only frontal-facing images were selected.

**Fig 1:**
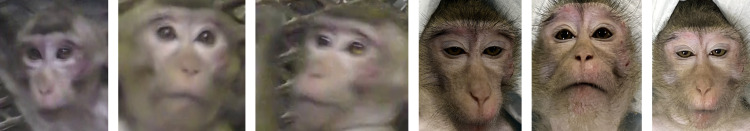
Examples of cropped facial images from a single monkey captured under varying lighting conditions, image quality, and poses.

For the second dataset, close-up images of monkeys under anesthesia (Ketamine) were obtained via the Sony Handycam. These images were taken multiple times with varying range: around 10 cm to 30 cm of distance exist between the monkey’s face and the camera, as shown in Fig 1. During the anesthesia, we also marked their bodies with non-permanent markers and then returned them to the group cage. After all of the monkeys regained consciousness, we recorded their movement inside the cage using the same Sony Handicam and included the recordings to the second dataset. Total number of classes and images utilized in the datasets are listed in Table 1.

**Table 1:** Number of images and corresponding classes across phases, grouped per dataset and housing.

Dataset 1 [Phase 1]		Dataset 2 [Phase 2 and Phase 3]					
		Housing #1		Housing #2		Housing #3	
ID	No. of Images	ID	No. of Images	ID	No. of Images	ID	No. of Images
1	55	1	149	1	140	1	169
2	21	2	144	2	146	2	171
3	66	3	149	3	148	3	171
4	28	4	148	4	182	4	144
5	58	5	148	5	148	5	158
6	27	6	148	6	176	6	157
7	69	7	147	7	149	7	131
8	60	8	148	8	166	8	163
		9	148	9	148	9	147
		10	113	10	158	10	152
		11	107	11	150	11	148
		12	121	12	147	12	143
				13	145	13	147
				14	145	14	156
				15	146	15	154
				16	135	16	96
						17	172

### Feature extraction

Various feature extraction and transformation algorithms were tested. Eigenface (EF)-based algorithm was utilized to reduce the dimensionality of grayscale images by converting them into column vectors and extracting significant features through projection into principal component dimensions. Local Binary Patterns Histograms (LBP-H), similar to techniques used in [33] were also tested as it was proven effective to capture texture information which are abundant in facial images. Scale Invariant Feature Transform (SIFT) [41] and Speeded up Robust Features (SURF) [42] when combined with Bag-of-words (BoW) model are some of the popular choices for object recognition and were also tested. In this study, the preprocessing step include resizing the input images into 300x200 pixels and convert them into grayscale by simple averaging across RGB channels.

### Eigenface algorithm

The Eigenface (EF) algorithm [9] is a principal component analysis (PCA)-based approach primarily used for face recognition tasks. It starts by normalizing the size and intensity of facial images, then converts these images into column vectors to form a high-dimensional image space. PCA is applied to this space to find the eigenvectors, also known as “eigenfaces” when related to facial images, which are the directions of maximum variance in the data. By projecting the original image vectors onto this lower-dimensional subspace, the algorithm effectively reduces dimensionality and captures the most significant features of the faces. This method emphasizes the global structure of the face, such as the overall shape and features configuration, rather than local details. In this study, principal components are retained until 90% cumulative contributions are achieved.

### Local binary patterns histograms

Local Binary Patterns Histograms (LBP-H) [11,40] is a texture descriptor used for image analysis, particularly effective in facial recognition due to its ability to capture fine-grained texture details. LBP operates by comparing each pixel with its surrounding neighbors, assigning a binary code that represents the pattern of neighboring pixels that are darker or lighter than the central pixel. This process is repeated for every pixel in the image, resulting in a texture descriptor for each pixel. The LBP histograms (LBP-H) are then computed by aggregating these descriptors over the image or sub-regions of the image, capturing the distribution of different patterns. This method is invariant to monotonic grayscale changes, making it robust for texture classification under varying lighting conditions.

### Scale invariant feature transform

The Scale Invariant Feature Transform (SIFT) [41] algorithm is designed to detect and describe local features in images. It is particularly robust to changes in scale, rotation, and illumination. SIFT works by identifying key points in an image, such as edges and corners, at multiple scale levels (often referred to as the “scale space”). For each key point, SIFT computes an orientation histogram based on the gradients of pixel intensities around the key point, creating a descriptor that captures the local image shape. These descriptors are invariant to image scale and rotation, and they provide robust matching across a substantial range of affine distortion, changes in 3D viewpoint, noise, and illumination changes. In this study, only the strongest five features were considered.

### Speeded up robust features

Speeded up robust features (SURF) [42] is an algorithm that improves upon SIFT by aiming to increase speed and efficiency while maintaining robustness. It relies on integral images for image convolutions, allowing it to compute features quickly and at different scales. SURF detectors identify points of interest from the determinant of the Hessian matrix and use the sum of the Haar wavelet response around the point of interest for orientation assignment. The SURF descriptor is then formed by computing the sum of the Haar wavelet response and the sum of the absolute values of the responses within a sliding orientation window, providing a unique and robust descriptor for feature matching. In this study, only the strongest three points were retained.

### Bag-of-words (BoW) model with SIFT and SURF

The Bag-of-Words (BoW) model, borrowed from text analysis, is applied to image recognition by treating image features as “words” [43]. When combined with SIFT or SURF, the descriptors extracted from these algorithms are quantized into a fixed-size vocabulary using clustering techniques like k-means [44], which are utilized in this study. Each image is then represented as a histogram of the frequency of each “visual word” in the vocabulary, essentially summarizing the distribution of feature descriptors within the image. This representation allows for the application of machine learning algorithms for tasks such as image classification and object recognition, leveraging the robust feature detection capabilities of SIFT and SURF. In this study, vocabulary size is fixed at 200.

### Classification models

Support Vector Machines (SVMs) [45] were evaluated with a variety of kernels including linear, polynomial of the 2nd and 3rd orders, and Radial Basis Function (RBF). Linear Discriminant Analysis (LDA) [46] was applied in its standard form, serving as a technique to reduce dimensionality and improve separability between classes. The k-Nearest Neighbors (kNN) algorithm [47,48] was also utilized, leveraging their simplicity and robustness for classification tasks. Whenever possible, parameter optimization was conducted via bayes optimization with maximum of 50 evaluations per parameter combination.

### Support vector machines

Support Vector Machines (SVMs) [45] are a powerful set of supervised learning methods used for classification, regression, and outlier detection. The core principle behind SVM is to find the hyperplane that best separates the different classes in the feature space. For non-linearly separable data, SVM uses kernel functions to map the input features into a higher-dimensional space where a linear separation is possible. The most common kernels are:

1. Linear kernel: It is the simplest form of SVM and works well when the data is linearly separable. The linear kernel is essentially just the dot product of two vectors, representing a straight line or hyperplane in the feature space.
2. Polynomial kernel: This kernel maps the original features into a polynomial feature space of specified degree (2nd, 3rd, etc.). It is useful for non-linear data and can model complex relationships by considering interactions between features up to the specified polynomial degree.
3. Radial basis function (RBF): The RBF kernel, often just referred to as the Gaussian kernel, is a popular choice for non-linear data. It adds flexibility by considering the distance between feature vectors in a more nuanced way, enabling the model to create more complex boundaries.

### Linear discriminant analysis

Linear Discriminant Analysis (LDA) [46] is both a dimensionality reduction technique and a linear classifier. As a dimensionality reduction method, LDA seeks to project the data onto a lower-dimensional space in such a way that maximizes the separation between multiple classes. It achieves this by finding the axes that maximize the distance between the means of different classes while minimizing the variance within each class. This makes LDA particularly effective for tasks where the primary goal is to achieve the best class separability.

### k-Nearest neighbors

The k-Nearest Neighbors (kNN) algorithm [47,48] is a simple, non-parametric method used for classification (and regression). kNN classifies a sample based on the majority class among its k nearest neighbors in the feature space. The distance between samples can be measured in various ways, commonly using Euclidean distance. kNN is robust to noisy training data and effective if the dataset is large. However, it can become computationally expensive as the dataset grows, and its performance depends significantly on the choice of k and the distance metric used.

### Parameter optimization with bayesian optimization

Parameter optimization, especially in complex models like SVM with different kernels, is crucial for achieving the best model performance. Bayesian optimization is an efficient strategy for global optimization of objective functions that are expensive to evaluate. It builds a probabilistic model of the function and uses it to make intelligent decisions about where to evaluate the function next. In the context of your models, Bayesian optimization was used to tune hyperparameters (like the regularization parameter in SVM, the degree of the polynomial kernel, or the number of neighbors in kNN) with a maximum of 50 evaluations per parameter combination. This approach helps in finding the optimal parameter values that lead to the best classification performance without exhaustively searching the entire parameter space, which can be time-consuming.

### Real-time processing and robustness on multiclass identification

A pooling and majority voting mechanism was introduced to stabilize the identification process across successive video frames, reducing the likelihood of inconsistent classifications. This approach aggregated classification results over a short time window, ensuring that the final identification was based on the most common outcome, making a table consisting of frames in rows and confidence of predicted classes in columns. These enhancements were crucial for the practical application of the facial recognition system in real-time scenarios, such as monitoring the behavior of cynomolgus monkeys in their natural habitats or controlled environments. In our system, we collected 0.5s worth of images before we classify them. Updates were also performed every 0.5s.

### Evaluation method

**Fig 2:**
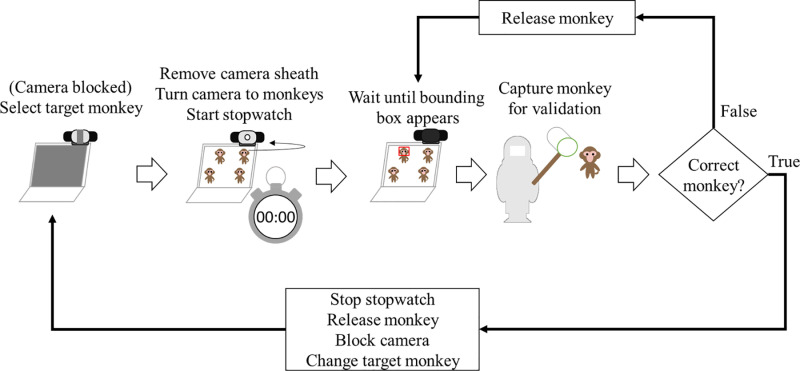
Protocol for real-time testing in the final evaluation phase. Each step demonstrates the process of identifying individual monkeys by detecting bounding boxes in a group setting.

Accuracy is chosen as the main metric for evaluation. All validations were of 10-fold cross validations, except during the real-testing in the final phase. In the real-testing phase, monkeys must be captured to confirm the recognition accuracy. As such, it is practically impossible to verify multiple monkeys at once and we opted to verify one at a time and use the following protocol instead (illustrated in Fig 2):

1. Change the recognition mode to only show bounding box for one selected monkey.
2. From ID 01 up to the last ID, one-by-one, do:
  Unblock the web camera, then point it upon the group of monkeys in the cage.
  When the bounding box shows up on a particular monkey, capture the detected monkey.
  Check the monkey’s ID on its thigh.
  If the monkey’s ID does NOT match with the currently selected ID, release the monkey and return to step 2a. Otherwise, release the monkey, block the camera, then change the selected ID to the next one.

An additional evaluation method by utilizing non-permanent markers was also performed. The protocol was largely similar to the above test, except without capturing/ releasing the monkeys. The monkey’s ID is written on their chest and painted their arms, thighs, and tails to color-code them. Color-coding the monkeys this way enables us to recognize monkeys even when their ID on their chest or thighs was not visible. The protocol for real-testing also prevented true negatives and false negatives from being recorded, making it impossible to compute the true ‘accuracy. As a result, alternative metrics such as number of captures and time needed until successful identification were utilized.

## Results

In the first phase of our study, we focused on the individual identification of cynomolgus monkeys using a variety of feature extraction methods and classification models. EF combined with Support Vector Machine (SVM) with Radial Basis Function (RBF) kernel emerged as the most effective, achieving high accuracy levels. This phase set the foundation for our system’s capability to accurately recognize individual monkeys in controlled single-monkey environments. Details are shown in Table 2.

**Table 2:** Results of 10-fold cross-validation for the first phase, showing accuracy (%) from various machine learning models versus different feature extraction methods.

*Machine Learning Model*	*Feature Extraction Method (Accuracy %)*			
	*LBP-H*	*EF*	*SIFT+BoW*	*SURF+BoW*
kNN	97.06	98.29	79.90	79.64
LDA	91.67	95.83	83.59	85.42
SVM	97.43	**97.65**	85.31	84.65

For the second phase, we expanded our system to recognize monkeys in group settings within large cages. The 10-fold cross-validation accuracies for the cages are: 88.10%, 95.67%, and 95.36% for housing 1, housing 2, and housing 3, respectively. In the third phase, we implemented the system in a near real-time context, introducing enhancements such as pooling and majority voting to address the challenges of real-time recognition. These enhancements were pivotal in maintaining consistent identification across successive frames, showcasing the system’s potential for real-world applications. Table 3 shows the result of the real-time recognition system. Monkey ID14 and 16 on housing 2 was unwell during the testing day and was not in the cage due to treatment and Table 4 show the results of the additional evaluation. During the time between the test with catch/release and the additional evaluation with non-permanent markers, three monkeys were transferred to other facility (ID11 and 13 from Housing #2; ID16 from Housing #3) and as such the evaluation was performed without them.

**Table 3:** Results of the final phase. Columns show the number of capture attempts required for correct identification and corresponding time duration (in minutes) across different housings. Averages (AVG) and standard deviations (SD) are provided.

Housing #1			Housing #2			Housing #3		
ID	Captures until correct [times]	Duration until correct [minutes]	ID	Captures until correct [times]	Duration until correct [minutes]	ID	Captures until correct [times]	Duration until correct [minutes]
1	2	3	1	1	2	1	1	2
2	1	1	2	5	18	2	10	33
3	6	13	3	7	26	3	2	4
4	6	15	4	1	3	4	6	11
5	1	2	5	5	28	5	8	22
6	2	5	6	10	34	6	12	39
7	4	14	7	6	19	7	5	16
8	3	9	8	3	8	8	3	8
9	1	3	9	7	14	9	1	6
10	9	22	10	8	22	10	8	22
11	1	3	11	3	9	11	3	6
12	2	4	12	9	21	12	2	6
			13	2	3	13	10	37
			14	–	–	14	4	13
			15	5	13	15	3	7
			16	–	–	16	12	34
						17	4	13
AVG	3.2	7.8	AVG	5.1	15.7	AVG	5.5	16.4
SD	2.6	6.7	SD	2.9	10.0	SD	3.8	12.5

**Table 4:** Results of the additional evaluation phase using non-permanent markers to color-code monkeys for identification, measured the time (in seconds) required for correct identification. Averages (AVG) and standard deviations (SD) are also shown.

Housing #1		Housing #2		Housing #3	
ID	Duration until correct [seconds]	ID	Duration until correct [seconds]	ID	Duration until correct [seconds]
1	20	1	59	1	69
2	27	2	9.5	2	112
3	25	3	61	3	40
4	12	4	69	4	16
5	3	5	2.3	5	44
6	32.5	6	36	6	13
7	33	7	43.7	7	53
8	34.5	8	36	8	45
9	15	9	43.7	9	28.5
10	58	10	47.3	10	94
11	59.5	11	–	11	42
12	17.5	12	60	12	5
		13	–	13	42
		14	62.3	14	66.5
		15	13.5	15	39
		16	17.5	16	–
				17	3
AVG	28.08	AVG	42.50	AVG	46.79
SD	16.37	SD	20.09	SD	30.64

## Discussion

The validation phase of our facial recognition system revealed notable performance metrics, particularly with the Eigenface (EF) combined with Support Vector Machine (SVM) approach, achieving an accuracy exceeding 80%. This level of accuracy is promising, indicating the potential efficacy of the system in real-world applications for the identification of cynomolgus monkeys in various settings.

Experts in our group usually require around 40 minutes (as a team of 2 persons or more) to 60 minutes per cage (when capturing alone), on average, to catch the correct monkey. During the real testing, the system also showed its usability as average time needed until correct monkey is identified reduced to around 15 minutes, which is significantly shorter. Table 4 is provided to show the ideal performance of the system – which can reach under 1 minute mark for correct identification. Expectedly, human catch-and-release is the slowest and costs the most time in this evaluation step.

However, it’s also important to acknowledge a significant limitation in the evaluation of our final phase: the reliance solely on precision (true positives divided by all positive predictions) for the final assessment. This metric, while valuable, does not provide a comprehensive view of the system’s performance, as it overlooks other critical aspects such as recall (true positives divided by the sum of true positives and false negatives) and the overall F1 score, which balances precision and recall. Nevertheless, the confusion matrix on the final phase must still be filled manually even if confirmation of multiple monkeys is possible.

We also focused our observation on specific monkeys—#10 in housing 1, #6 and #12 in housing 2, and #2, #6, #13, and #16 in housing 3—which during the real-time test, highlighted varying degrees of system performance. We found that Monkey #10 in housing 1 exhibited the lowest precision during the training phase although the precision rate is still above 80%. This discrepancy underscores the challenges in achieving consistent recognition accuracy across different subjects, potentially due to varying facial features, expressions, or environmental factors within the cages. Monkey #16 in housing 3 presented a unique challenge due to the limited sample size available for validation, which raises concerns about the robustness of our findings for this particular subject. The reduced data points could lead to overfitting or unreliable performance metrics, indicating a need for further data collection to ensure a more reliable analysis. Other monkeys performed relatively well during the training and reduction in classification during real testing might be reasoned due to the lower image quality from the webcam compared to the handycam utilized for data collection.

Despite these challenges, the system demonstrated reasonable performance during training sessions for the majority of the subjects. This success suggests that with further refinement, particularly in addressing the identified limitations and expanding the dataset, the system could significantly improve in accuracy and reliability.

In light of these findings, future work should focus on enhancing the system’s adaptability to diverse environmental conditions and subject variabilities. This could involve integrating more sophisticated machine learning algorithms capable of handling complex data variations or developing more robust data augmentation techniques to increase the diversity of training samples. Alternatively, other facilities that do not have high turnover and do not require minimizing learning dataset may consider other acquisition strategies (for example, treat-motivated acquisition) and timings. In case which environmental conditions can be minimized and facial images can be regularly obtained, robust data augmentation might even be unnecessary, and implementation of incremental machine learning techniques may also be considered.

## Conclusion

Our comprehensive study has successfully established a novel automatic facial recognition system specifically designed for cynomolgus monkeys. The system has shown remarkable proficiency, particularly when employing the Eigenface (EF) method in conjunction with the Support Vector Machine (SVM) equipped with the Radial Basis Function (RBF) kernel. This combination has achieved an accuracy rate of 97.65% in 10-fold cross validation. When focusing on individual monkeys under group housing conditions, correct identification can be achieved under 20 minutes on average.

The introduction of real-time processing enhancements, such as pooling and majority voting, has been pivotal in overcoming the inherent challenges associated with dynamic and real-time recognition scenarios. These advancements are instrumental in maintaining consistent and reliable identification, further illustrating the system’s robustness and its potential applicability in real-world contexts: automatic identification while minimizing potential stress caused to animals. Though, it must be admitted that our current setup still uses classical marking (anesthesia and tattoo) to check correct identification. We hope that future systems may no longer need such setup.

Despite encountering certain limitations, such as the reliance on precision as the sole evaluation metric in the final phase and challenges with specific subjects due to limited sample sizes or environmental factors, the system has demonstrated considerable promise. The observed discrepancies and challenges underscore the necessity for ongoing refinement, particularly in expanding the dataset and enhancing the system’s adaptability to a wider range of environmental conditions and subject variabilities.

Looking forward, our future endeavors will concentrate on refining multi-face recognition capabilities, with a particular focus on optimizing the majority voting method to enhance performance further. Additionally, we aim to explore the system’s applicability and potential contributions to broader fields, including primate research, conservation efforts, and possibly extending to other species, thereby broadening the horizon of biometric identification technologies in wildlife studies.

In conclusion, this study not only contributes a valuable tool to the field of primatology but also opens avenues for future research and applications that could significantly benefit conservation efforts and enhance our understanding of primate behavior and social dynamics.
